# Schisandrin B Alleviates Renal Tubular Cell Epithelial–Mesenchymal Transition and Mitochondrial Dysfunction by Kielin/Chordin-like Protein Upregulation via Akt Pathway Inactivation and Adenosine 5′-Monophosphate (AMP)-Activated Protein Kinase Pathway Activation in Diabetic Kidney Disease

**DOI:** 10.3390/molecules28237851

**Published:** 2023-11-29

**Authors:** Weilin Liu, Fan Li, Dongwei Guo, Congyuan Du, Song Zhao, Juan Li, Zhe Yan, Jun Hao

**Affiliations:** 1 Department of Pathology, Hebei Medical University, Shijiazhuang 050017, Chinaguo1270674313@163.com (D.G.);; 2 Hebei Key Laboratory of Kidney Diseases, Shijiazhuang 050017, China; 3 Center of Metabolic Diseases and Cancer Research, Institute of Medical and Health Science of Hebei Medical University, Shijiazhuang 050017, China; 4 Department of Infectious Diseases, Fourth Hospital of Hebei Medical University, Shijiazhuang 050011, China; 5 Department of Nephrology, Second Hospital of Hebei Medical University, Shijiazhuang 050000, China

**Keywords:** schisandrin B, KCP, TGF-β1, PGC-1α, epithelial–mesenchymal transition, mitochondrial dysfunction, diabetic kidney disease

## Abstract

Diabetic kidney disease is a common complication of diabetes and remains the primary cause of end-stage kidney disease in the general population. Schisandrin B (Sch B) is an active ingredient in Schisandra chinensis. Our study illustrates that Sch B can mitigate renal tubular cell (RTC) epithelial–mesenchymal transition (EMT) and mitochondrial dysfunction in db/db mice, accompanied by the downregulation of TGF-β1 and the upregulation of PGC-1α. Similarly, Sch B demonstrated a protective effect by reducing the expression of TGF-β1, α-SMA, fibronectin, and Col I, meanwhile enhancing the expression of E-cadherin in human RTCs (HK2 cells) stimulated with high glucose. Moreover, under high glucose conditions, Sch B effectively increased mitochondrial membrane potential, lowered ROS production, and increased the ATP content in HK2 cells, accompanied by the upregulation of PGC-1α, TFAM, MFN1, and MFN2. Mechanistically, the RNA-seq results showed a significant increase in KCP mRNA levels in HK2 cells treated with Sch B in a high glucose culture. The influence of Sch B on KCP mRNA levels was confirmed by real-time PCR in high glucose-treated HK2 cells. Depletion of the KCP gene reversed the impact of Sch B on TGF-β1 and PGC-1α in HK2 cells with high glucose level exposure, whereas overexpression of the KCP gene blocked EMT and mitochondrial dysfunction. Furthermore, the PI3K/Akt pathway was inhibited and the AMPK pathway was activated in HK2 cells exposed to a high concentration of glucose after the Sch B treatment. Treatment with the PI3K/Akt pathway agonist insulin and the AMPK pathway antagonist compound C attenuated the Sch B-induced KCP expression in HK2 cells exposed to a high level of glucose. Finally, molecular autodock experiments illustrated that Sch B could bind to Akt and AMPK. In summary, our findings suggested that Sch B could alleviate RTC EMT and mitochondrial dysfunction by upregulating KCP via inhibiting the Akt pathway and activating the AMPK pathway in DKD.

## 1. Introduction

Diabetic kidney disease (DKD) is the most prevalent factor that causes chronic kidney disease (CKD), significantly impacting the quality of life of patients and even resulting in death [1]. The accumulating evidence has revealed that a variety of factors contribute to the development of DKD, including hyperglycemia, abnormal lipid metabolism, oxidative stress, inflammation, aberrant autophagy, and aging [2,3,4]. As a result, renal cells, such as mesangial cells, renal tubular cells (RTCs), podocytes, and endothelial cells, are damaged by these detrimental factors, leading to various morphological changes, such as mesangial region expansion, renal interstitial fibrosis, and podocyte apoptosis [5,6].

In these pathological changes, renal interstitial fibrosis is associated with the epithelial–mesenchymal transition (EMT) of RTCs. The epithelial marker of RTCs (E-cadherin) gradually disappears, while the mesenchymal marker (α-SMA) slowly appears [7]. Consequently, RTCs synthesize and secrete various components of the extracellular matrix (ECM) such as fibronectin (FN) and collagen I, eventually resulting in interstitial fibrosis [8]. Mechanistically, the transforming growth factor-beta 1 (TGF-β1) signaling pathway is the most critical fibrosis-regulating pathway involved in the EMT of RTCs in DKD [9].

In recent years, mitochondrial dysfunction in the kidney has increasingly garnered attention and was reported to perform a critical function in the pathogenesis of DKD [10]. Given the high oxygen consumption of the kidney, it is particularly susceptible to mitochondrial dysfunction, which can lead to cell autophagy, apoptosis, and necrosis due to the overproduction of reactive oxygen species (ROS) and low levels of ATP during the development and progression of DKD [11]. Peroxisome proliferator-activated receptor-gamma coactivator-1α (PGC-1α) was first identified as a partner of peroxisome proliferator-activated receptor (PPAR) and is known to regulate a series of active mitochondrial genes by binding to numerous transcription factors [12]. A decrease in expression of PGC-1α has been demonstrated in various animal DKD models [13]. The activation of PGC-1α and its downstream targets, including mitochondrial transcription factor 1 (TFAM) and nuclear respiratory factor 1 (NRF1), have been shown to reduce renal injury in DKD by improving mitochondrial function and reducing ROS levels [14]. Additionally, mitochondria-targeted antioxidants, such as mitoTEMPO and elamipretide, have been reported to improve DKD by decreasing mitochondrial ROS production [15].

*Schisandrin B* (*Sch B*) is the main natural compound of Schisandra chinensis, a kind of traditional Chinese herb. Sch B possesses diverse pharmacological functions such as antioxidation, anti-inflammation, and anti-tumor effects [16]. It is reported that Sch B could alleviate T2DM by promoting insulin release through the GLP-1R/cAMP/PKA signaling pathway [17]. Song et al. discovered that Sch B could prevent the kidney damage caused by diabetes and restore its function [18]. Also, Sch B was reported to protect the kidneys in DKD by suppressing the inflammatory response and oxidative stress [19]. Despite this, the precise mechanism is still not well known.

Kielin/chordin-like protein (KCP) is a type of secretory protein that possesses cysteine-rich chordin repeats and is known to enhance bone morphogenetic protein (BMP) signaling and inhibit the TGF-β1 signaling pathway [20]. The PI3K/Akt and AMPK pathways are two critical pathways involved in maintaining cell growth, nutrition metabolism, proliferation, and migration. These pathways may be activated by the phosphorylation of Akt at Ser 473 and AMPK at Thr 172 [21]. Throughout the process of DKD-related pathogenesis and progression, PI3K/Akt pathway over-activation and AMPK inactivation were proven to mediate the injury to RTCs [22,23].

In this research, we aimed to show that Sch B mitigates EMT and mitochondrial dysfunction in RTCs of db/db mice. Likewise, Sch B was found to suppress the TGF-β1 pathway and EMT while enhancing the PGC-1α pathway and mitochondrial function in high glucose-treated HK2 cells. RNA-seq and cellular experiments indicated that KCP is a downstream target of Sch B, which was increased by Sch B, contributing to the downregulation of the TGF-β1 pathway and upregulation of PGC-1α. Then, we showed that the PI3K/Akt pathway was inactivated and the AMPK pathway was activated by Sch B in HK2 cells subjected to high glucose levels. The activation of PI3K/Akt pathway or inactivation of AMPK pathway affected the effect of Sch B on KCP, TGF-β1, and PGC-1α. Finally, a molecular autodock experiment revealed the binding between Sch B and Akt and AMPK.

## 2. Results

### 2.1. Schisandrin B Attenuates Epithelial–Mesenchymal Transition of Renal Tubular Cells in db/db Mice

**Figure 1:**
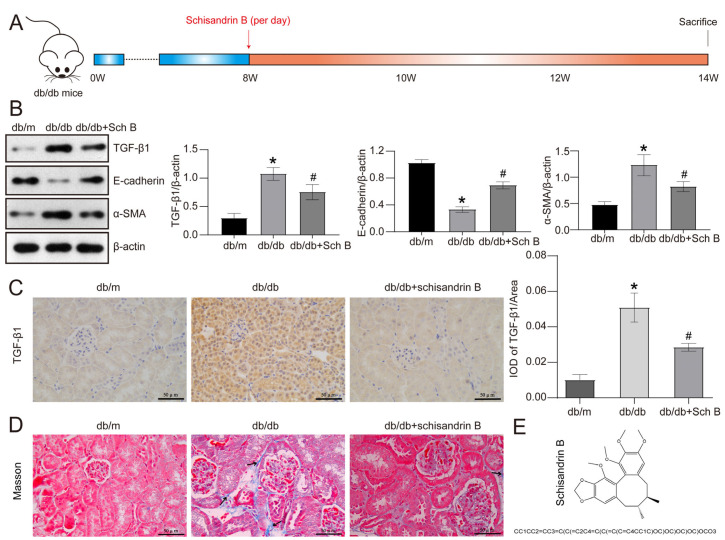
Schisandrin B attenuates epithelial–mesenchymal transition of renal tubular cells in db/db mice. (**A**) Type II diabetes mellitus db/db mice were administered with schisandrin B. (**B**) Western blot of TGF-β1, E-cadherin, and α-SMA expression in the kidneys of db/m mice, db/db mice, and db/db mice administered with schisandrin B. (**C**) Immunohistochemistry of TGF-β1 in the kidneys of db/m mice, db/db mice, and db/db mice administered with schisandrin B. (**D**) Masson staining of the kidneys of db/m mice, db/db mice, and db/db mice administered with schisandrin B. (**E**) Two-dimensional molecular structure and Simplified Molecular-Input Line-Entry System (SMILES) structure of schisandrin B. * *p* < 0.05 versus db/m mice, # *p* < 0.05 versus db/db mice. IOD: Integrated Optical Density.

We initially investigated the impact of Sch B (Figure 1E) on the kidneys of the db/db mouse, a type II diabetes mouse model (Figure 1A). The results demonstrated a substantial elevation in urinary albumin in db/db mice in contrast with db/m mice, which was reversed by Sch B treatment (*p* < 0.05). Furthermore, serum creatinine was elevated by 2.0 times in db/db mice relative to db/m mice, but this elevation was effectively decreased by the Sch B treatment (*p* < 0.05) (Table 1). Nevertheless, when comparing db/db mice treated with Sch B to untreated db/db mice, we found no remarkable variations in body weight, food intake, daily urinary volumes, Cystatin C, blood urea nitrogen, or blood glucose levels, kidney weight/body weight, or kidney weight (Table 2). Additionally, the Western blotting analysis demonstrated an upregulation of the EMT-related markers TGF-β1 and α-SMA and a reduction in the levels of E-cadherin in the kidneys of db/db mice in contrast with db/m mice, which were reversed by Sch B treatment. Specifically, the expression levels of α-SMA and TGF-β1 were reduced by 29.9% and 33.3%, respectively, while the E-cadherin level was elevated by 2.1 times following treatment with Sch B (Figure 1B). Furthermore, the IHC results indicated that TGF-β1 primarily localized in the cytoplasm of RTCs, and its expression was inhibited by Sch B treatment in db/db mice (Figure 1C). Again, Masson trichrome staining illustrated a significant increase in ECM accumulation in the renal interstitium of db/db mice relative to db/m mice, which was suppressed by treatment with Sch B (Figure 1D).

### 2.2. Schisandrin B Alleviates Mitochondrial Dysfunction of Renal Tubular Cells from db/db Mice

**Figure 2:**
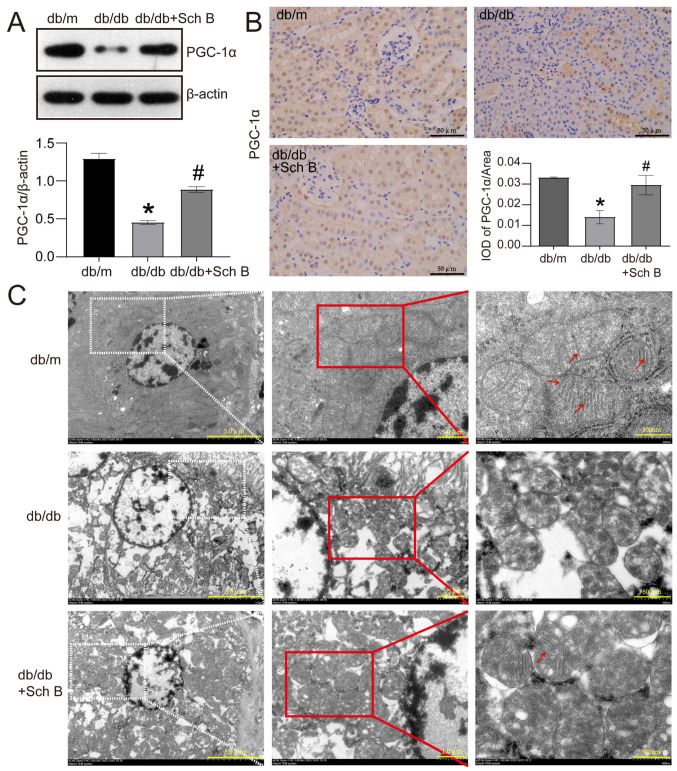
Schisandrin B alleviates mitochondrial dysfunction of renal tubular cells in db/db mice. (**A**) Western blot of PGC-1α expression in the kidneys of db/m mice, db/db mice, and db/db mice administered with schisandrin B. (**B**) Immunohistochemistry of PGC-1α expression in the kidneys of db/m mice, db/db mice, and db/db mice administered with schisandrin B. (**C**) Electron microscopy observation of the renal tubular cells of db/m mice, db/db mice, and db/db mice administered with schisandrin B. * *p* < 0.05 versus db/m mice, # *p* < 0.05 versus db/db mice. IOD: Integrated Optical Density.

The maintenance of intracellular mitochondrial homeostasis is crucial for ensuring proper cellular function. Therefore, we conducted further investigations to explore the impact of Sch B on mitochondrial function in the kidneys of db/db mice using Western blot analysis, IHC, and electron microscopy. Western blotting showed that the mitochondrial homeostasis regulator, PGC-1α, was reduced by 65.0% in the kidneys of db/db mice relative to db/m mice. However, this reduction was effectively reversed by the administration of Sch B (*p* < 0.05) (Figure 2A). The immunohistochemistry results, shown in Figure 2B, confirmed that the PGC-1α level was lower in the RTCs of db/db mice in contrast with db/m mice. Nonetheless, Sch B treatment was found to reverse the downregulation of PGC-1α induced by diabetes mellitus. Additionally, the electron microscopy observations revealed that the matrix granules of mitochondria disappeared, and mitochondrial cristae were fused and unclear in the RTCs of db/db mice, compared to those of db/m mice. These phenomena were partially improved in Sch B-treated db/db mice (Figure 2C).

### 2.3. Schisandrin B Inhibits Epithelial–Mesenchymal Transition in HK2 Cells Exposed to Elevated Levels of Glucose

**Figure 3:**
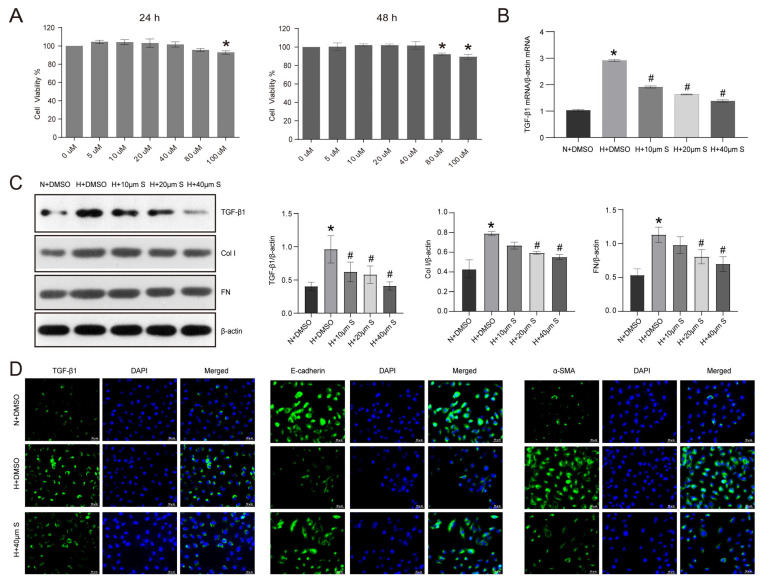
Schisandrin B inhibits epithelial–mesenchymal transition in high glucose-treated HK2 cells. (**A**) Cell viability of normal glucose-cultured HK2 cells treated with different concentrations of schisandrin B for 24 and 48 h. * *p* < 0.05 versus 0 μM schisandrin B group. (**B**) Real-time PCR for TGF-β1 mRNA in HK2 cells treated with schisandrin B. (**C**) Western blot of TGF-β1, Col I, and FN in HK2 cells treated with schisandrin B. (**D**) Immunofluorescence of TGF-β1, E-cadherin, and α-SMA in HK2 cells of N + DMSO group, H + DMSO group, and H + 40 μM schisandrin B group. * *p* < 0.05 versus N + DMSO group, # *p* < 0.05 versus H + DMSO group. N: normal glucose, H: high glucose, FN: fibronectin, Col 1: human collagen type I.

To further clarify the impact and mechanism of Sch B on RTCs in DKD, we cultured the HK2 cell line in a medium containing high glucose and treated it with varying concentrations of Sch B. As depicted in Figure 3A, the cell viability experiment indicated that 100 μmol/L Sch B had a cytotoxic effect at 24 h, while both 80 μmol/L and 100 μmol/L Sch B had a cytotoxic effect at 48 h in normal glucose-exposed HK2 cells. Consequently, we used Sch B concentrations lower than 80 μmol/L to treat HK2 cells. Subsequently, the RT-PCR findings showed that the expression of EMT-associated TGF-β1 at the mRNA level was increased upon high glucose treatment in HK2 cells (Figure 3B). However, when treated with 10, 20, and 40 μmol/L Sch B, the TGF-β1 mRNA level was decreased by 34.3%, 44.1%, and 52.6%, respectively, compared to DMSO treatment in high glucose-treated HK2 cells (Figure 3B). Similarly, the Western blotting results revealed that the high glucose-induced increase in TGF-β1 protein levels was ameliorated by Sch B treatment (Figure 3C). Specifically, the TGF-β1 protein level was, respectively, decreased by 35.4%, 39.6%, and 57.3% upon Sch B treatment compared to the DMSO treatment. Additionally, the expression of the ECM components collagen I and FN was enhanced by high glucose treatment, which was effectively reversed with Sch B treatment (Figure 3C). Furthermore, immunofluorescence was used to evaluate the expression of α-SMA, TGF-β1, and E-cadherin in high glucose-treated HK2 cells subjected to 40 μmol/L Sch B. The results indicated that Sch B significantly inhibited TGF-β1 and α-SMA expression and upregulated E-cadherin expression in high glucose-treated HK2 cells (Figure 3D).

### 2.4. Schisandrin B Improves Mitochondrial Dysfunction in HK2 Cells Exposed to Elevated Levels of Glucose

**Figure 4:**
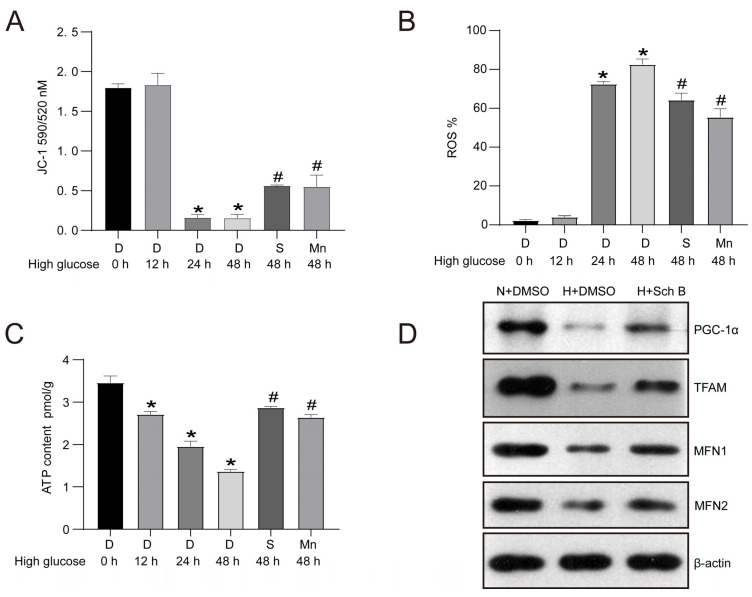
Schisandrin B improves mitochondrial function in high glucose-treated HK2 cells. (**A**) Mitochondrial membrane potential detection by JC-1 staining in high glucose-cultured HK2 cells treated with schisandrin B. (**B**) ROS detection of high glucose-cultured HK2 cells treated with schisandrin B. (**C**) ATP content detection in high glucose-cultured HK2 cells treated with schisandrin B. (**D**) Western blot of PGC-1α, TFAM, MFN1, and MFN2 expression in HK2 cells of N + DMSO group, H + DMSO group, and H + schisandrin B group. * *p* < 0.05 versus 0 h group, # *p* < 0.05 versus D 48 h group. D: DMSO, S: schisandrin B, Mn: MnTBAP, N: normal glucose, H: high glucose.

Firstly, the effect of Sch B on MMP was investigated in HK2 cells by staining with JC-1. As illustrated in Figure 4A, the high glucose treatment contributed to a remarkable reduction in MMP in HK2 cells. MMP was reduced by 91.1% and 91.4% after 24 and 48 h of high glucose stimulation, respectively, in contrast with the 0 h glucose treatment group (*p* < 0.05). Additionally, similar to the MnTBAP, Sch B also exhibited a protective effect on mitochondria and increased MMP by 3.7 fold. The accumulation of ROS is a hallmark of mitochondrial dysfunction. An intracellular ROS content assay revealed that 24 and 48 h of high glucose treatment significantly increased ROS in HK2 cells (Figure 4B). However, both Sch B and MnTBAP decreased ROS content in high glucose-treated HK2 cells (Figure 4B). As mitochondria are the main organelle for ATP production, we also assessed the influence of Sch B on the intracellular ATP content in HK2 cells that were subjected to high glucose levels. As expected, the high glucose treatment decreased ATP production in HK2 cells relative to cells that were exposed to normal glucose levels. However, the Sch B treatment increased the ATP content by 2.10 times in 48 h in HK2 cells subjected to high glucose levels (*p* < 0.05) (Figure 4C). Furthermore, the Western blotting analysis showed that the mitochondrial quality control-associated proteins PGC-1α, mitochondrial transcription factor A (TFAM), mitofusin 1 (MFN1), and mitofusin 2 (MFN2) were remarkably reduced in HK2 cells of the high glucose group in contrast with those of the normal glucose group. However, Sch B treatment increased the expression of PGC-1α, MFN1, TFAM, and MFN2 (Figure 4D).

### 2.5. KCP Upregulation Mediates Schisandrin B-Regulated Epithelial–Mesenchymal Transition and Mitochondrial Dysfunction in HK2 Cells Exposed to High Glucose Levels

**Figure 5:**
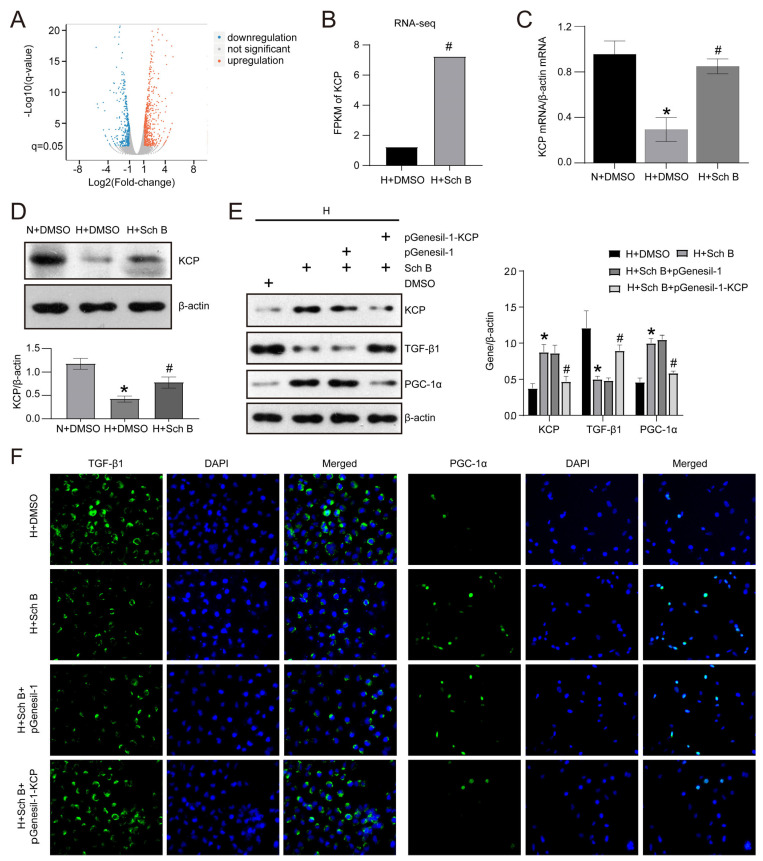
KCP upregulation mediates schisandrin B−regulated EMT and mitochondrial dysfunction in high glucose−treated HK2 cells. (**A**) RNA-seq of high glucose−cultured HK2 cells treated with schisandrin B. (**B**) RNA-seq of KCP expression in high glucose-cultured HK2 cells treated with schisandrin B. (**C**) Real-time PCR of KCP mRNA in HK2 cells treated with schisandrin B. (**D**) Western blot of KCP protein expression in HK2 cells treated with schisandrin B. * *p* < 0.05 versus N + DMSO group, # *p* < 0.05 versus H + DMSO group. (**E**) Western blot of KCP, TGF-β1, and PGC-1α expression in HK2 cells with KCP knockdown. (**F**) Immunofluorescence of TGF-β1 and PGC-1α in HK2 cells transfected with pGenesil-1-KCP plasmid. * *p* < 0.05 versus H + DMSO group, # *p* < 0.05 versus H + Sch B + pGenesil-1 group. N: normal glucose, H: high glucose.

We utilized RNA-seq to detect the differentially expressed genes (DEGs) following Sch B treatment in HK2 cells subjected to high glucose levels to explain the exact mechanism responsible for the improvement in EMT and mitochondrial dysfunction by Sch B. Compared to H + DMSO, the H + Sch B group displayed 444 upregulated and 307 downregulated DEGs (q value < 0.05, fold change > 2 or <−2) (Figure 5A). As illustrated in Figure 5B, KCP was one of the top 10 DEGs, which was significantly enhanced by Sch B in HK2 cells exposed to high glucose. Moreover, the RT-PCR analysis confirmed that the expression of KCP at the mRNA level was decreased in high glucose-treated HK2 cells compared to cells cultured with normal glucose levels, and Sch B treatment significantly increased KCP mRNA expression (Figure 5C). Similarly, the Western blotting analysis demonstrated that KCP protein expression was reduced by 64.1% in HK2 cells exposed to high glucose levels relative to control cells (*p* < 0.05). Subsequently, Sch B caused a 1.8-fold increase in KCP protein expression in cells exposed to high glucose levels (*p* < 0.05) (Figure 5D). Additionally, we inhibited KCP expression in HK2 cells exposed to high glucose levels by transfecting the cells with a KCP-specific shRNA plasmid and effectively attenuated Sch B-induced suppression of TGF-β1 and promotion of PGC-1α expression (Figure 5E). Similar to the findings of the Western blotting, the immunofluorescence (IF) assay also demonstrated that the depletion of KCP improved the influence of Sch B on TGF-β1 and PGC-1α in HK2 cells subjected to high glucose levels (Figure 5F).

**Figure 6:**
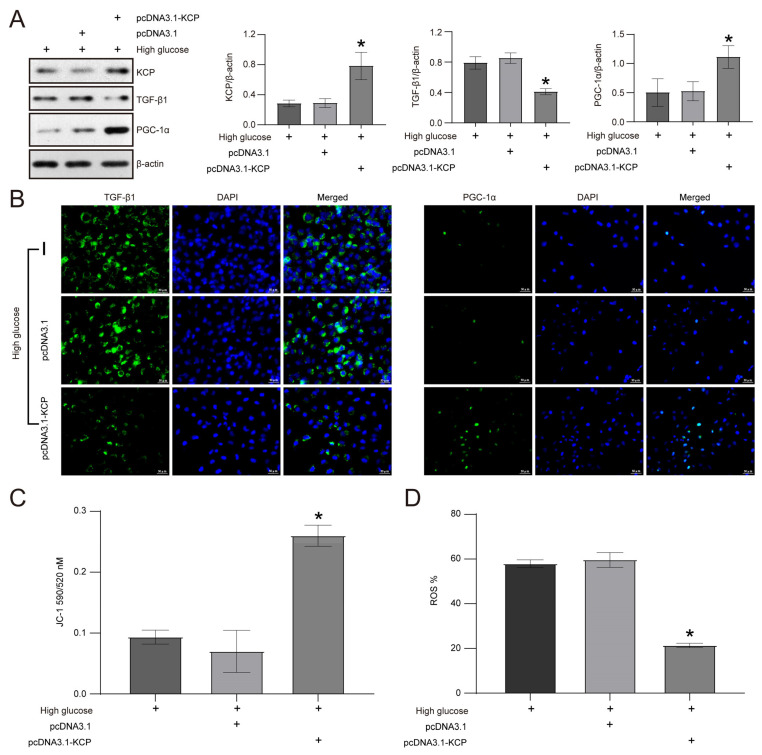
Overexpression of KCP decreases the expression of TGF-β1 and increases the expression of PGC-1α in high glucose-cultured HK2 cells. (**A**) Western blot of KCP, TGF-β1, and PGC-1α expression in HK2 cells with KCP overexpression. (**B**) Immunofluorescence of TGF-β1 and PGC-1α in high glucose-cultured HK2 cells transfected with pcDNA3.1-KCP plasmid. (**C**) Mitochondrial membrane potential detection by JC-1 staining in high glucose-cultured HK2 cells transfected with pcDNA3.1-KCP plasmid. (**D**) ROS detection of high glucose-cultured HK2 cells transfected with pcDNA3.1-KCP plasmid. * *p* < 0.05 versus pcDNA3.1 group.

Conversely, the overexpression of KCP in HK2 cells subjected to high glucose levels inhibited TGF-β1 and increased PGC-1α expression (Figure 6A). Specifically, the TGF-β1 protein levels were decreased by 51.9% with pcDNA3.1-KCP transfection compared to pcDNA3.1 transfection in HK2 cells exposed to high glucose levels. In contrast, compared to the high glucose-treated HK2 cells transfected with pcDNA3.1, the PGC-1 expression level was increased by 2.1-fold in cells transfected with pcDNA3.1-KCP (*p* < 0.05) (Figure 6A). Similar to the findings of the Western blotting, the IF analysis revealed that pcDNA3.1-KCP transfection reduced TGF-β1 expression and enhanced PGC-1α expression in HK2 cells subjected to high glucose levels (Figure 6B). Additionally, JC-1 staining demonstrated that the MMP of high glucose-treated HK2 cells was markedly enhanced by KCP overexpression (Figure 6C). Similarly, ROS content was decreased by 64.2% with pcDNA3.1-KCP transfection compared to pcDNA3.1 transfection in HK2 cells subjected to high glucose levels (*p* < 0.05) (Figure 6D).

### 2.6. PI3K/Akt Pathway Inhibition and AMPK Pathway Activation Are Involved in Schisandrin B-Induced Increase in KCP Expression in HK2 Cells Exposed to High Glucose Levels

**Figure 7:**
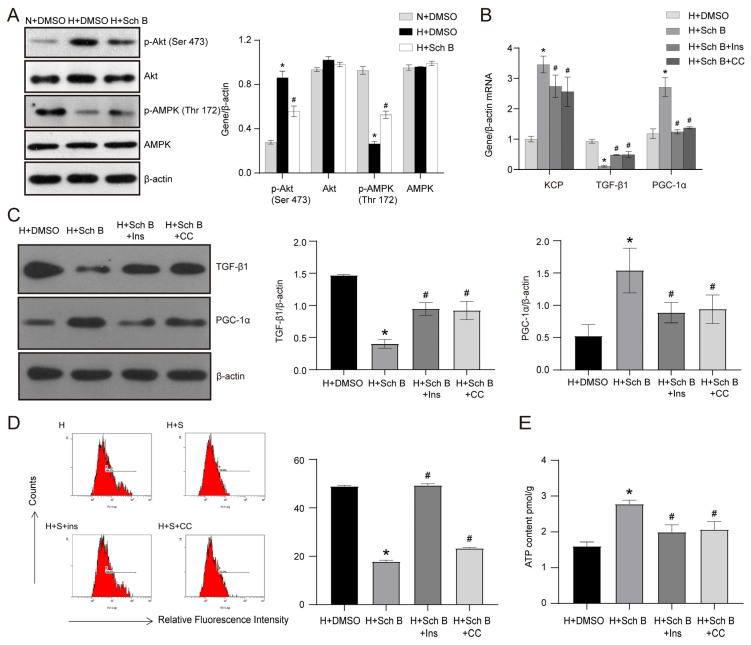
PI3K/Akt pathway inhibition and AMPK pathway activation are involved in the schisandrin B-induced increase in KCP expression in high glucose-treated HK2 cells. (**A**) Western blot of the effect of schisandrin B on Akt and AMPK in HK2 cells. * *p* < 0.05 versus N + DMSO group, # *p* < 0.05 versus H + DMSO group. (**B**) Real-time PCR of KCP, TGF-β1, and PGC-1α mRNA in schisandrin B-stimulated HK2 cells treated with insulin (Ins) and compound C (CC). (**C**) Western blot of TGF-β1 and PGC-1α in schisandrin B-stimulated HK2 cells treated with Ins and CC. (**D**) ROS detection of schisandrin B-stimulated HK2 cells treated with Ins and CC. (**E**) ATP content detection of schisandrin B-stimulated HK2 cells treated with Ins and CC. * *p* < 0.05 versus H + DMSO group, # *p* < 0.05 versus H + Sch B group. N: normal glucose, H: high glucose.

The hyperactivation of the PI3K/Akt pathway and the inactivation of the AMPK pathway was found to mediate the pathogenesis and development of DKD. We conducted further experiments to elucidate the potential roles of the PI3K/Akt and the AMPK pathways in Sch B-induced KCP expression in HK2 cells exposed to high glucose levels. As depicted in Figure 7A, the Sch B treatment effectively prevented high glucose-induced Akt phosphorylation at Ser 473 and reversed the downregulation of phosphorylated AMPK at Thr 172. Subsequently, the high glucose-treated HK2 cells were treated with a PI3K/Akt pathway agonist (insulin) and an AMPK pathway antagonist (compound C) to assess their effect on Sch B-induced changes in the expression of TGF-β1 and PGC-1α at the mRNA level. Figure 7B shows that insulin and compound C partially reversed the effects of Sch B on TGF-β1 and PGC-1α mRNA expression in HK2 cells exposed to high levels of glucose. Similar to the findings of the RT-PCR, the Western blot analysis revealed that insulin and compound C increased TGF-β1 expression by 2.3-fold in HK2 cells exposed to high glucose plus Sch B (*p* < 0.05) (Figure 7C). Furthermore, the PGC-1α protein levels were reduced by 42.4% and 39.0% with insulin and compound C treatment, respectively (*p* < 0.05) (Figure 7C). Furthermore, the intracellular ROS content assay revealed that, in high glucose-treated HK2 cells, Sch B inhibition of ROS was enhanced by insulin and compound C (Figure 7D). Specifically, the ROS content was elevated by 2.8-fold and 1.3-fold in the high glucose + Sch B + insulin group and high glucose + Sch B + compound C group compared to the high glucose + Sch B group (Figure 7D). Finally, the ATP content assay showed that insulin and compound C weakened the stimulatory influence of Sch B on ATP production in HK2 cells cultured in high glucose (Figure 7E).

### 2.7. Schisandrin B Could Bind with Akt at Ser 473 Phosphorylation Site and AMPK at Thr 172 Phosphorylation Site

**Figure 8:**
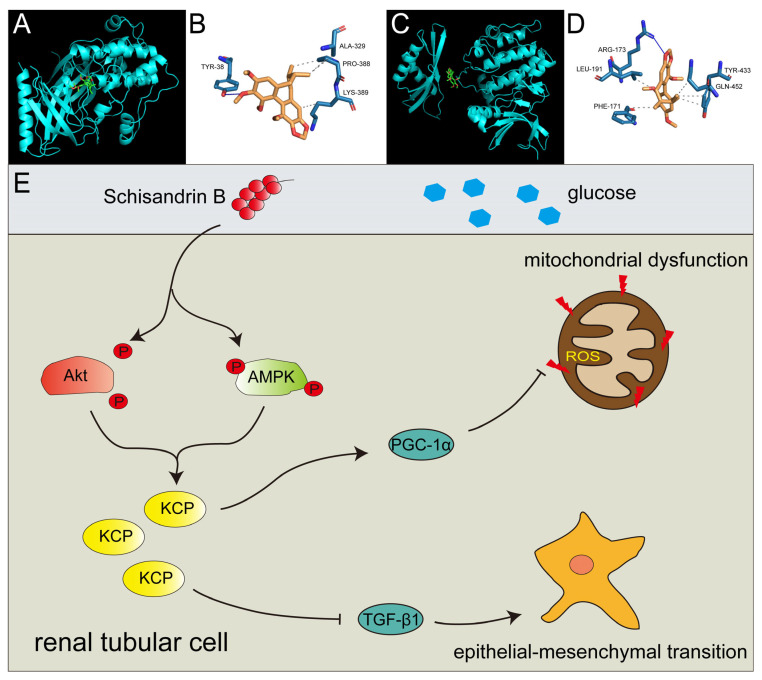
Schisandrin B could bind with Akt and AMPK in the mechanism of schisandrin B’s effect on renal tubular cells in diabetes mellitus. (**A**) Molecular docking of schisandrin B and Akt. (**B**) The binding of schisandrin B to Akt. (**C**) Molecular docking of schisandrin B and AMPK. (**D**) The binding of schisandrin B to AMPK. (**E**) Model of the regulation of schisandrin B on Akt, AMPK, KCP, TGF-β1, PGC-1α, EMT, and mitochondrial function in RTCs of diabetic kidney disease.

As micromolecules typically affect cell function by binding to critical protein regions, we hypothesized that Sch B might bind to the active sites of both Akt and AMPK. A molecular docking analysis using AutoDock Vina 1.1.2 software demonstrated that Sch B could bind to both proteins. Specifically, Sch B bound to the Akt protein at the region containing Tyr 38, Ala 329, Pro 388, and Lys 389 (Figure 8A,B) and to the AMPK protein at the region containing Tyr 433, Gln 452, Arg 173, Leu 191, and Phe 171 (Figure 8C,D). We hypothesized that Sch B would alter the phosphorylation state of the Thr 172 site on AMPK since Thr 172 of AMPK was located in this region. For Akt, we postulated that the structural change of Pro 388 and Lys 389 due to Sch B binding might affect the phosphorylation of Ser 473.

## 3. Discussion

This study confirmed that Sch B alleviates EMT in RTCs in diabetes mellitus by effectively blocking the TGF-β1 pathway, along with a consequent elevation in the α-SMA level and reduction in the E-cadherin level. Similarly, Sch B also exhibited an anti-EMT function in tumor cells. Li et al. showed that Sch B inhibits cell migration, invasion, and EMT in cancer stem-like cells derived from H661, NCI-H460, and large-cell lung cancer cells. Mechanistically, the effect of Sch B on EMT is mediated through the suppression of the NF-κB and P38 MAPK pathways [24]. Similarly, Sch B significantly inhibits lung and bone metastasis of murine breast cancer cells (4T1) by suppressing EMT [25]. Furthermore, in the UUO mice model of renal fibrosis, Sch B markedly decreases the expression of collagen IV and vimentin in the kidney, and the levels of pro-fibrotic transcription factors, including Slug and Zeb2, are also reduced [26]. These above findings suggest that Sch B could inhibit the advancement of DKD by inhibiting the EMT of RTCs.

In addition, we demonstrated that Sch B improved mitochondrial dysfunction in RTCs exposed to a hyperglycemic environment, reducing ROS production and restoring the ATP content in Sch B-treated HK2 cells. Han et al. similarly reported that Sch B treatment improves oxidative stress and MMP depolarization in angiotensin II-stimulated rat aortic endothelial cells, through the regulation of the keap1/Nrf2 signaling pathway [27]. Guo et al. discovered that schisandrin A, B, and C inhibit mitochondrial ROS generation and ATP release, as well as IL-1β secretion in THP-1 cells infected with P. acnes [28]. Additionally, Wang et al. reported that Sch B attenuates osteoporosis, inhibits the stimulation of the MAPK and NF-κB pathways, and scavenges ROS via Nrf2 signaling in mice with ovariectomy-induced bone loss [29]. Therefore, Sch B was suggested to ameliorate mitochondrial dysfunction in RTCs of DKD.

To investigate the specific mechanism underlying Sch B inhibition of EMT and mitochondrial dysfunction, we used RNA-seq to screen for DEGs in Sch B-treated HK2 cells. We identified an increase in KCP expression in Sch B-treated HK2 cells and subsequently confirmed that KCP was capable of suppressing EMT and improving mitochondrial function. Soofi et al. reported similar results, showing that the overexpression of KCP in kidneys reduces the expression of fibrosis-related markers, such as collagen IV and α-SMA, in UUO mice and improves survival in a folic acid-induced acute kidney injury (AKI) model [30]. They also demonstrated that secreted KCP functions as a modulator of the TGF-β superfamily pathway, inhibiting both the TGF-β and activin-A signaling pathways [31]. Overall, our findings demonstrate that KCP is a promising downstream target of Sch B for inhibiting EMT and improving mitochondrial function in RTCs of DKD.

PI3K/Akt pathway over-activation and AMPK pathway inactivation have been reported to mediate the pathogenesis of DKD [32]. In contrast, the suppression of the PI3K/Akt pathway or activation of AMPK with metformin has been shown to protect renal cells in DKD [22,33]. Herein, we demonstrated that Sch B restrained Akt phosphorylation and promoted AMPK phosphorylation, resulting in the upregulation of KCP in the RTCs of DKD mice. Consistent with our findings, Wang et al. reported that, in osteosarcoma cells, SaOS2 and U2OS, the PI3K/Akt pathway was regulated by Sch B. Additionally, Sch B suppresses cell viability and migration and triggers cell apoptosis via the PI3K/Akt pathway [34]. Similarly, Zhao et al. showed that Sch B attenuates H/R-induced H9c2 cell injury by activating the AMPK/Nrf2 pathway [35]. We also performed molecular autodock experiments for Sch B and Akt and AMPK, and revealed that Sch B could bind to the phosphorylation site-containing region of AMPK. Thus, our data suggest that Sch B-induced upregulation of KCP is facilitated by the suppression of the PI3K/Akt pathway and sensitization of the AMPK pathway in RTCs of DKD mice.

## 4. Materials and Methods

### 4.1. Materials

The primary antibodies utilized in this investigation were as follows: primary antibody against KCP (TA326631) from OriGene Technology Co., Ltd. (Rockville, MD, USA); primary antibodies against TGF-β1 (21898-1-AP), E-cadherin (20874-1-AP), Col 1 (14695-1-AP), fibronectin (66042-1-Ig), α-SMA (14395-1-AP), MFN1 (13798-1-AP), MFN2 (67487-1-Ig), PGC-1α (66369-1-Ig), TFAM (22586-1-AP), Akt (10176-2-AP), and phospho-Akt (Ser 473) (66444-1-Ig) from Proteintech Group, Inc. (Wuhan, Hubei, China); and primary antibodies against phospho-AMPK (AF3423) and AMPK (AF6423) from Affinity Biologicals Inc. (Ohio, USA). An antibody against β-actin (TA-09), horseradish peroxidase (HRP)-conjugated secondary antibodies (ZB2305 and ZB2301), an immunohistochemistry kit (SP9000), and Alexa Fluor 488-conjugated goat anti-rabbit secondary antibody (ZF-0511) were procured from Zhongshan Golden Bridge Technology Co. (Beijing, China). RIPA lysis buffer was procured from Solarbio (Beijing, China). 4′, 6-diamidino-2-phenylindole (DAPI) was procured from Boster (Wuhan, Hubei, China). TransGen Biotech Co. (Beijing, China) provided the TransIntro EL Transfection Reagent (FT201-01). The HiFiScript gDNA Removal cDNA Synthesis Kit (CW2582M) was purchased from Kangwei Century Biotechnology Co., Ltd. (Taizhou, Jiangsu, China). MonAmp™ SYBR^®^ Green qPCR Mix (MQ00101S) was purchased from Monad Biotechnology Inc. (Wuhan, Hubei, China). A Trizol solution (15596-026) was obtained from Ambion, Thermo Fisher Scientific Inc. (Waltham, MA, USA). Working Weigert’s Iron Haematoxylin Solution, Ponceau acid fuchsin solution, phosphomolybdic–phosphotungstic acid, and aniline blue solution were purchased from Zhuhai Boson Biotechnology Co., Ltd. (Guangdong, China). Schisandrin B (61281-37-6) was purchased from Shanghai Ronghe Medical Technology Development Co. (Shanghai, China). Sigma Chemical Co. (St. Louis, MO, USA) supplied the insulin (#I2643). MCE CO. (Monmouth Junction, NJ, USA) supplied the compound C. Jingsai Co. (Wuhan, Hubei, China) supplied the pGenesil-1 plasmid for the construction of the shRNA plasmid. The target sequence of the KCP shRNA plasmid (pGenesil-1-KCP) was CCTACCACAGCCAAGTGTATG. The KCP expression plasmid (pcDNA3.1-KCP) was purchased from YouBio Co. (Changsha, Hunan, China). MnTBAP was purchased from APExBIO Technology LLC. (Houston, TX, USA). Nanjing Jiancheng Bioengineering Institute. (Nanjing, Jiangsu, China) supplied the 7′-dichlorofluorescein-diacetate (DCFH-DA). The mitochondrial membrane potential assay kit with JC-1 and ATP content test kit were from Biyuntian Co. (Shanghai, China). Baso Diagnostics Inc. (Zhuhai, Guangdong, China) supplied the Masson trichrome stain kit.

### 4.2. Animal Experiment

Changzhou Cavens Experimental Animal Co. Ltd. provided sixteen db/db mice and eight db/m mice aged 8 weeks old (Changzhou, Jiangsu, China). db/db mice are a strain of mice with a defect in the Leptin receptor gene, also known as Leprdb/db mice. db/m mice were used as controls and for breeding; here, “m” represents the misty mutant gene. All animal experimental protocols were approved by the Institutional Animal Care and Use Committee at Hebei Medical University (Approval Code: IACUC-Hebmu-P 2023068). The mice were placed in controlled conditions with a 12 h light/dark cycle and unrestricted access to a standard chow diet and water. Sixteen db/db mice were classified into two groups at random, vehicle or Sch B (Sch B). The method for preparing the Sch B suspension was as follows: dissolve 20 mg of Sch B in 4 mL of a 1% sodium carboxymethyl cellulose solution to achieve a concentration of 5 mg/mL. The mice in the Sch B group were administered Sch B via oral gavage (50 mg/kg weight per day), whereas the mice in the vehicle group received an equivalent volume of carboxymethylcellulose sodium. After six weeks, blood and urine were collected for biochemical detection. Sodium pentobarbital (120 mg/kg weight) was used to euthanize the mice, and kidney tissues were removed for comparative analysis.

### 4.3. Cell Culture 

Dulbecco’s Modified Eagle Medium/Nutrient combination F-12 Ham (DMEM/F12 3:1 combination) with 10% serum, 100 units per mL penicillin, and 100 g per mL streptomycin were used to culture the human renal proximal tubular epithelial cell line (HK2) at 37 °C and 5% CO_2_. For in vitro tests, cells with a confluence of roughly 70–80% were employed. Cells were serum starved for 12 h to prepare the cells for further treatment.

To investigate how Sch B affects EMT, KCP expression, Akt, and the AMPK pathway of HK2 cells subjected to high glucose levels, the HK2 cells were separated into three groups at random: normal glucose (5.5 mM) (N + DMSO), high glucose (30 mM) (H + DMSO), and high glucose with Sch B (H + Sch B). To investigate how Sch B affects mitochondrial dysfunction, HK2 cells grown under increased glucose conditions were stimulated with DMSO (0.1%), Sch B (40 μmol/L), and MnTBAP (100 μmol/L) for 48 h. To further understand the effects of KCP knockdown, the cells grown in high glucose levels were randomized into four groups: H + DMSO, H + Sch B, high glucose plus Sch B, and pGenesil-1 (H + Sch B + pGenesil-1) and high glucose together with Sch B and pGenesil-1-KCP (H + Sch B + pGenesil-1-KCP). To determine the effect of KCP overexpression on EMT and mitochondrial dysfunction, HK2 cells grown in high glucose were separated into three groups at random: the untransfected group (-), pcDNA3.1 transfection group (pcDNA3.1), and pcDNA3.1-KCP transfection group (pcDNA3.1-KCP). The HK2 cells were serum starved for 12 h to prepare the cells for further treatment. The HK2 cells were randomized into H + DMSO, H + Sch B, high glucose plus Sch B and insulin (2 μg/mL) (H + Sch B + Ins), and high glucose plus Sch B and compound C (10 μmol/L) (H + Sch B + CC) groups and cultivated in high glucose. In the H + Sch B + Ins group and the H + Sch B + CC group, the cells were pre-stimulated with insulin and compound C for 2 h, and then cultivated in high glucose. The influence of the treatments on the PI3K/Akt and AMPK pathways in each group were examined.

### 4.4. Cell Transfection

Cell transfection was conducted using TransIntro EL Transfection Reagent (TransGen Biotech, FT201-01, Beijing, China) as per the directions stipulated by the manufacturer. Specifically, 4 μg of plasmid diluted in 200 µL Opti-MEM Medium (Thermo Fisher Scientific, 31985062, Waltham, MA, USA) was mixed with 6 µL TransIntro EL at ambient temperature. After 20 min, the mixture was introduced to HK2 cells in 6-well plates. The cells were collected 48 h after plasmid transfection for Western blotting, immunofluorescence, intracellular ROS assays, ATP content assays, and JC-1-mitochondrial membrane potential assays.

### 4.5. Western Blotting

The kidneys of diabetic and normal mice or HK2 cells were used to extract total protein before being cultured with the indicated treatment using RIPA lysis buffer at 4 °C for 30 min. The supernatant after centrifugation was obtained, and the protein concentrations were assessed using the BCA technique. The same amount of protein was then electro-transferred to PVDF membranes after being loaded on an SDS-PAGE gel for electrophoresis. The membranes were pre-incubated with 5% BSA at 37 °C for 1 h to block any non-specific binding and then incubated overnight at 4 °C with a primary antibody diluted in TTBS (0.2 M Tris, pH 7.4, 1.5 M NaCl, 0.1% thimerosol, and 0.5% Tween 20) containing 1% BSA. The next morning, the blots were incubated with a secondary antibody conjugated with HRP for 2 h at ambient temperature after being rinsed thrice in TTBS. Subsequently, the bands were visualized using an ECL solution. The relative protein expression was measured by normalizing to the housekeeping gene β-actin.

### 4.6. Immunohistochemistry

Renal sections were used for the detection of TGF-β1 and PGC-1α by immunohistochemistry. Specifically, the sections were placed in a 65 °C incubator for 6 h, followed by defatting in xylene three times for 10 min each. Then, the sections were rehydrated with 100%, 95%, 80%, 75%, and 50% alcohol for 5 min each, after which the antigen was extracted using a pressure cooker in citrate buffer (10 mM, pH 6.0). Afterward, 3% hydrogen peroxide was applied to the portions to prevent peroxidase activity. To prevent non-specific binding, the sections were pretreated with goat serum and incubated at 37 °C. Then, the sections were incubated with primary antibodies overnight at 4 °C. Following the rinsing of the samples in PBS, they were treated for 30 min with biotin-labeled goat secondary antibody at 37 °C and another 30 min with HRP-conjugated streptavidin at 37 °C. The positive signals were then visualized using 3, 3′-diaminobenzidine tetrahydrochloride (DAB). Thereafter, the protein expression was analyzed using the integrated optical density (IOD) of positive signals. For the negative control, PBS was used in place of the primary antibody.

### 4.7. Immunofluorescence

HK2 cells were seeded onto coverslips and cultured with the indicated treatment. The HK2 cells were fixed with 4% paraformaldehyde (PFA) for 20 min before permeabilizing the cells for 10 min at ambient temperature with 0.3% Triton X-100. After blocking with goat serum for 30 min, the HK2 cells were incubated with primary antibody at 4 °C overnight. The primary antibodies utilized and their corresponding dilution ratios were as follows: TGF-β1 (1:50), α-SMA (1:100), and E-cadherin (1:100). The sections were washed in PBS and then incubated with Alexa Fluor 488-conjugated goat secondary antibody and 4′, 6-diamidino-2-phenylindole (DAPI) for 2 h at 37 °C. After that, images were taken and used for analysis using an inverted fluorescence microscope (CX31, Olympus Corporation, Tokyo, Japan).

### 4.8. Intracellular ROS Assay

The ROS levels of the HK2 cells were quantified using the DCFH-DA Cellular ROS Detection Assay Kit (Jiangsu, China) and a flow cytometer in the FITC channel. Specifically, after washing with cold PBS, the HK2 cells were collected and re-suspended in the diluted DCFH-DA with serum-free medium (SFM, 10 μmol/L DCFH-DA). After incubation at 37 °C for 20 min, the HK2 cells were rinsed in SFM three times and resuspended with 500 μL SFM, followed by detection using a flow cytometer (FC 500, Beckman Coulter, Laguna Woods, CA, USA).

### 4.9. ATP Content Assay

Intracellular ATP was measured utilizing an ATP assay kit from Shanghai Beyotime Biotechnology Co., LTD (Shanghai, China). As directed by the manufacturer, the HK2 cells were treated with lysis buffer and rinsed in PBS before centrifugation at 14,000× *g* rpm for 5 min to obtain the supernatant. Thereafter, the ATP content was determined by mixing the supernatant with the ATP detection solution. Later, utilizing a monochromator microplate reader (Fluoroskan FL, Thermo Fisher Scientific), the luminescence was measured. The standard curve was used to compute the ATP content in the samples, which was then normalized to the protein concentration.

### 4.10. JC-1-Mitochondrial Membrane Potential Assay

The JC-1 staining kit (Beyotime Biotechnology, Shanghai, China) was adopted to measure the mitochondrial membrane potential (MMP). In brief, the HK2 cells exposed to the specified treatments were rinsed in PBS. The HK2 cells were subsequently treated with the JC-1 dye, and the cells were cultured at 37 °C for 20 min in darkness. The HK2 cells were analyzed using a flow cytometer (FC 500, Beckman Coulter, Laguna Woods, CA, USA) after they were washed.

### 4.11. Cell Viability Detection

MTS reagents [3-(4,5-dimethylthiazol-2-yl)-5(3-carboxymethoxyphenyl)-2-(4-sulfopheny)-2H-tetrazolium,inner salt] (Promega Co. Madison, WI, USA.) were used to determine cell viability. Specifically, 20 μL of MTS reagent was added to HK2 cells seeded in a 96 well plate. After 4 h, the absorbance was obtained utilizing a microplate reader at 490 nm. The percentage of control cells was used to calculate the relative cell viability.

### 4.12. Electron Microscopy

The ultra-microstructure of the mouse kidney tissues was examined by transmission electron microscopy (TEM). Specifically, 2% glutaraldehyde diluted by PBS was used to fix the samples at 4 °C overnight. After the samples were washed with PBS, the secondary fix was performed using 2% osmium tetroxide. Ultrathin sections were created after the samples were dried and embedded, stained with 2% uranyl acetate and lead citrate, and examined using a transmission electron microscope (HT7700, Hitachi, Japan).

### 4.13. RNA-Seq

Total RNA was extracted from HK2 cells of the high glucose group and the high glucose plus Sch B (40 μmol/L) group. After the RNA was purified and fragmented, first and second-strand cDNA syntheses were performed. In turn, the cDNA was adenylated and amplified using the polymerase chain reaction (PCR) method, followed by the ligation of an adapter to the end of the cDNA fragment. Finally, sequencing was conducted using an Illumina NovaSeq 6000 (Illumina, San Diego, CA, USA).

### 4.14. Real-Time PCR

Following the prescribed methods, a conventional real-time polymerase chain reaction (RT-PCR) was performed. Briefly, the total RNA from HK2 cells was isolated with the use of a Trizol solution. Reverse transcription using the HiFiScript gDNA Removal cDNA Synthesis Kit yielded complementary DNA (cDNA). The MonAmp™ SYBR^®^ Green qPCR Mix was utilized to perform qRT-PCR for 40 cycles. The PCR settings were: 95 °C for 15 min, followed by 40 cycles of 95 °C for 10 s, 56 °C for 30 s, and 72 °C for 30 s. To identify the specificity of amplification, melting curves were generated. Table 2 lists the primers, with β-actin serving as an internal reference. Relative gene expression was estimated utilizing the 2^−∆∆Ct^ method. All experiments were carried out three times separately.

### 4.15. Molecular Autodock Analysis

The two-dimensional structure of Sch B was derived from the PubChem database (https://pubchem.ncbi.nlm.nih.gov, accessed on 20 February 2023) for molecular docking. The AMPK (6c9ga) and Akt (7nh5) protein structure files were obtained from the PDB platform (https://www.rcsb.org, accessed on 20 February 2023). Sch B, AMPK, and Akt were molecularly docked using the AutoDock Vina program. The Protein-Ligand Interaction Profiler (PLIP) (https://projects.biotec.tu-dresden.de/plip-web/plip/index, accessed on 20 February 2023) website was utilized for visualizing the binding sites of Sch B, AMPK, and Akt.

### 4.16. Masson Trichrome Staining

Renal fibrosis and ECM accumulation were visualized in mouse samples using Masson trichrome staining. Briefly, after deparaffinizing and rehydrating the sections, Working Weigert’s Iron Haematoxylin Solution was used to stain the sections for 10 min. The sections were then stained for 10 min with the Ponceau acid fuchsin solution, followed by a wash with 1% glacial acetic acid. Subsequently, phosphomolybdic–phosphotungstic acid was used for differentiation before subjecting the samples to 3 min of staining in an aniline blue solution. The sections were then washed with 1% glacial acetic acid; after that, the sections were washed with ddH2O, covered with an acetic acid solution for 5 min, and then dehydrated in 95% ethanol for 2–3 s and in 100% ethanol for 2–3 s. The preparations were then air-dried and mounted and covered with coverslips.

### 4.17. Statistical Analysis

The data are reported as the mean ± standard deviation (SD) from a minimum of three independent experiments. For the statistical analyses, we utilized GraphPad Prism 8 (Version 8, GraphPad Software). In comparisons involving two groups, the student’s t-test was utilized. For comparisons involving more than two groups, one-way analysis of variance (ANOVA) and Bonferroni’s post hoc test were utilized. The Kruskal–Wallis test was utilized to analyze the non-normally distributed data. *p* < 0.05 was set as the significance threshold.

## 5. Conclusions

Taken together, this study suggests that Sch B alleviates RTC EMT and mitochondrial dysfunction by KCP upregulation via Akt pathway inactivation and AMPK pathway activation in diabetic kidney disease (Figure 8E).

## Figures and Tables

**Table 1:** Physical characteristics and biochemical parameters of mice.

Characteristic	db/m	db/db	db/db + Sch B
Body weight (g)	25.60 ± 0.55	55.60 ± 1.66 *	50.24 ± 4.06
Food intake (g/day)	3.81 ± 0.24	9.96 ± 0.22 *	9.35 ± 0.46
Daily urinary volume (mL)	0.33 ± 0.13	4.10 ± 0.48 *	4.15 ± 0.84
Kidney weight (g)	0.14 ± 0.008	0.18 ± 0.011 *	0.19 ± 0.016
Kidney weight/body weight (%)	0.56 ± 0.032	0.32 ± 0.027 *	0.38 ± 0.054
Blood glucose (mmol/L)	6.25 ± 0.42	29.43 ± 2.17 *	23.48 ± 5.74
Serum creatinine (μmol/L)	8.68 ± 0.33	17.33 ± 4.92 *	8.73 ± 0.83 #
Blood urea nitrogen (mmol/L)	5.90 ± 0.35	8.35 ± 0.29 *	7.41 ± 0.67
Cystatin C (mg/L)	0.28 ± 0.05	0.34 ± 0.04	0.28 ± 0.10
Urine albumin excretion (μg/24 h)	17.0 ± 6.85	516.76 ± 60.91 *	237.68 ± 49.29 #

* *p* < 0.05 versus db/m group, # *p* < 0.05 versus db/db group.

**Table 2:** PCR primers for KCP, TGF-β1, PGC-1α, and β-actin.

	Forward Primer	Reverse Primer	Product
Human KCP	GGGACACCAGTATCAGAGCCA	CCCCATCTTGACAGACGCAG	245 bp
Human TGF-β1	AGCAACAATTCCTGGCGATAC	CTAAGGCGAAAGCCCTCAAT	137 bp
Human PGC-1α	ACACTTTGCGCAGGTCAAAC	AGCAGGGTCAAAGTCATCTGAG	116 bp
Human β-actin	AAGGCCAACCGCGAGAA	ATGGGGGAGGGCATACC	183 bp

## Data Availability

All data in this study are available from the corresponding author upon reasonable request.
